# Upregulation of *ica* Operon Governs Biofilm Formation by a Coagulase-Negative *Staphylococcus caprae*

**DOI:** 10.3390/microorganisms11061533

**Published:** 2023-06-09

**Authors:** Hilla Oknin, Yulia Kroupitski, Moshe Shemesh, Shlomo Blum

**Affiliations:** 1 Institute for Postharvest Technology and Food Sciences, Department of Food Science, Agricultural Research Organization, Volcani Institute, Rishon LeZion 7505101, Israel; 2 Department of Bacteriology and Mycology, Kimron Veterinary Institute, Rishon LeZion 7534503, Israel

**Keywords:** CoNS, biofilm formation, extracellular matrix, sub-clinical mastitis, PIA, dairy food safety

## Abstract

*Staphylococcus caprae* is a Gram-positive, coagulase-negative staphylococci (CoNS), which appears as commensal in the skin, as well as a prevalent mastitis pathogen of goats. Occasionally, it is also associated with infections in humans. Biofilm formation has been identified as a putative virulence factor in *S. caprae*. Biofilms are multicellular communities protected by a self-produced extracellular matrix (ECM), which facilitates the resistance of bacterial cells to antimicrobial treatments. The ECM is constructed by exopolysaccharides, including the major exopolysaccharide—polysaccharide intercellular adhesion (PIA), regulated by the *ica* operon in *Staphylococcus* species. The aim of this study was to characterize the expression of the *ica* operon in relation to biofilm formation in *S. caprae*. Results showed that within a few hours of growth, *S. caprae* could adhere to polystyrene surfaces, start to accumulate, and form biofilm. Peak biofilm biomass and maturation were reached after 48 h, followed by a reduction in biomass after 72 h. Confocal laser scanning microscopy showed the expression of matrix-associated proteins and polysaccharides at various time points. The expression dynamics of the *ica* operon were investigated using real-time reverse transcriptase PCR (RT)-qPCR, which showed elevated expression during the early stages of biofilm formation and subsequent downregulation throughout the biofilm aging process. In conclusion, our results show that the *ica* operon is essential in regulating biofilm formation in *S. caprae*, similar to other *Staphylococcus* species. Furthermore, the robustness of the observed biofilm phenotype could account for the successful intramammary colonization and may explain disease persistence caused by this pathogenic bacterium.

## 1. Introduction

A Gram-positive bacterium, *Staphylococcus caprae*, first described by Devriese et al. in 1983, is a commensal species of coagulase-negative staphylococci (CoNS) found widely on the skin and the mammary glands of goats [1]. Since then, it has been increasingly shown that *S. caprae* is a prevalent mastitis pathogen of goats, leading to persistent sub-clinical infections through causing significant economic losses in the dairy industry [1,2,3,4]. Furthermore, this bacterium appears to be an emerging human pathogen, often associated with bone and joint infections and sometimes invasive infections, with multi-drug resistance being reported [5,6]. Notable virulence factors detected in *S. caprae* include fibronectin-binding and the formation of persistent biofilms [7].

Biofilm formation is a virulence factor that may facilitate adherence and colonization, immune evasion, and resistance to antimicrobial treatments of CoNS in the mammary gland [8,9,10]. Biofilm is defined as a multicellular bacterial community that grows on various surfaces or interfaces embedded within a self-produced polymeric matrix [11]. Biofilm formation has been described as a successful survival strategy for many bacterial species in most natural environments. The biofilm mode of growth can highly protect bacteria from stress conditions, such as UV damage, acid exposure, salinity, pH gradients, and desiccation, can host immune defense, and increases resistance to antibiotics or disinfectants [8,12,13,14]. Biofilm formation is a complex and multi-stage biological process typically consisting of three phases: adhesion, maturation, and detachment. In addition to being a physical shelter against fluctuating environmental conditions, the polymeric matrix also contributes to adhesion to the associated surface and helps recruit nutrients and minerals for the entire biofilm community [11]. Besides, it appears that most crucial biological processes are highly coordinated through an intra and intercellular signaling network within the multicellular community. Such arrangement enables bacterial cells accurately sense their immediate environment for establishing a thriving community in highly hostile and competitive ecological niches. The process of environmental sensing is usually mediated through various signaling molecules that generally govern the type and robustness of biofilm formed by different organisms. On the other hand, mature biofilm-embedded bacteria can be dispersed at some point from the biofilm to look for a new substrate(s) for forthcoming successful establishment [15].

Biofilm formation mostly depends on the synthesis of an extracellular matrix that holds the constituent cells together. Certain exopolysaccharides, including the major exopolysaccharide PIA (polysaccharide intercellular adhesin) or PNAG (poly-N-acetylglucosamine), play an important role in biofilm accumulation and maturation. The extracellular matrix (ECM) is constructed by PIA, extracellular DNA (eDNA), and surface proteins (cell wall-anchored proteins). These exopolymeric substances often surround and protect embedded bacteria [16]. Thus, biofilm bacteria are often more resistant to various antimicrobials due to this protective layer [17]. In *Staphylococcus* species, the PIA production is regulated by the *ica* operon (intercellular adhesion), which includes the *icaADBC* open reading frames with genes *icaA*, *icaD*, *icaB*, and *icaC*. The expression of the *ica* operon is influenced by various stress conditions, such as extreme temperatures, antibiotics, anaerobic conditions, and osmolarity [18]. The *icaA* gene product is a transmembrane protein involved in synthesizing poly-N-acetylglucosamine polymer and requires the *icaD* gene product for optimal activity [19]. The *icaA* and *icaD* products work together to produce N-acetyl-glucosamine oligomers that can reach a maximum length of 20 residues when co-expressed with *icaC* [18,20]. Besides, the *icaC* product is responsible for the translocation of the developing polysaccharide to the bacterial surface, while the *icaB* product is involved in the deacylation of the poly-N-acetylglucosamine polymer and translocation of the polymer to the outer surface of bacterial cell [18,21]. It was therefore hypothesized that the *ica* operon could play a significant role in regulating exopolysaccharide production and subsequent biofilm formation by CoNS, such as *S. caprae*. Accordingly, the motivation of the study was to investigate the role of *ica* operon during biofilm formation by a pathogen associated with chronic mastitis in goats and sporadic infections in humans. Thus, we aimed to characterize the biofilm formation process of *S. caprae*. Furthermore, the study investigated the dynamics of *ica* operon expression during biofilm formation. Due to the conservation of the *ica* operon and its crucial role in regulating biofilm formation by several *Staphylococcus* species, it is believed that understanding the functionality and involvement of this operon in persistency by under-characterized CoNS such as *S. caprae* is of utmost interest.

## 2. Materials and Methods

### 2.1. Bacterial Strains and Culture Conditions

The *Staphylococcus caprae* strain GBY4178 was isolated from a case of chronic mastitis in a goat and is part of the Kimron Veterinary Institute mastitis—CoNS collection (Bet-Dagan, Israel). Bacterial species were identified by API Staph32 (bioMérieux, Marcy-l’Étoile, France) and by Matrix-assisted laser desorption/ionization-time of flight (MALDI-TOF) mass spectrometry (MS) (Bruker Daltonics, Billerica, MA) following the manufacturer’s protocols. The strain was routinely grown on blood agar plates (Merck, Darmstadt, Germany). For biofilm formation, a starter culture was first grown to a stationary phase in Lysogeny broth (LB) medium at 37 °C in a shaking incubator, resulting in approximately 1 × 10^9^ cells/mL. Ten μL of the bacterial suspension was then mixed with 3 mL of tryptic soy broth (TSB) (Difco Labs, Detroit, MN, USA) supplemented with 2.5% lactose in 12-well polystyrene plates (Barnaor, Israel) and incubated at 37 °C under static conditions for different time periods (3 h, 7 h, 17 h, 24 h, 32 h, 48 h and 72 h) to examine biofilm formation dynamics.

### 2.2. Preparation of Lactose

A 50% lactose stock solution (Difco Labs, Detroit, MN, USA) was prepared in distilled deionized water (DDW) and sterilized with a 0.2 µm filter (Millex, Merck, Darmstadt, Germany). The lactose stock was diluted in autoclaved TSB to a final concentration of 2.5% (*w*/*v*).

### 2.3. Detection of Bacterial Adherence

The bacterial adherence to the surface was determined using a 12-well polystyrene plate (Bar-Naor, Petah Tikva, Israel). Ten μL of bacterial suspension were mixed with 3 mL of TSB containing 2.5% lactose and seeded in each well. The plate was incubated at 37 °C under static conditions for different times (0 h–8 h, 16 h, 17 h, 24 h, 32 h, 48 h, and 72 h). Viable bacteria were quantified after washing the wells twice with phosphate-buffered saline (PBS) solution, dislodging the adhered bacteria by scraping and re-suspending in PBS. The biofilms generated at different time points (16 h, 17 h, 24 h, 32 h, 48 h, and 72 h) were subjected to mild sonication with an Ultrasonic processor (Sonics, Newtown, CT, USA) for 20 s (amplitude, 20%; pulse, 10 s; pause, 10 s). After vortexing, the culture was serially diluted, plated on LB agar, and colonies were counted. The biofilm samples were also stained with 0.1% crystal violet (CV; Merck, Darmstadt, Germany) following a previously described procedure [22]. Briefly, the wells were washed twice with DDW, stained with crystal violet, incubated for 20 min, washed twice with DDW, and dried overnight. The crystal violet was then eluted with 33% acetic acid for 30 min, and the absorbance was measured at 595 nm using a spectrophotometer.

### 2.4. Biofilm Characterization

To assess the robustness of formed biofilms at different time points, the FilmTracerTM LIVE/DEAD Biofilm Viability Kit (Molecular Probes, Eugene, OR, USA) was used. The washed biofilms were treated with the staining reagents for 20 min, allowing for differentiation between live and dead cells. The green SYTO 9 dye stained live bacteria, while the red PI dye stained dead bacteria. The fluorescence emission from the stained samples was measured using an SP8 CLSM (Leica, Wetzlar, Germany) equipped with 488 and 552 nm lasers. The depth of the biofilm was analyzed by generating optical sections at 5 μm intervals, as previously described [17,23]. The composition of the biofilm was studied using CLSM with two dyes: FilmTracer SYPRO Ruby Biofilm Matrix stain (Invitrogen, Paisley, UK), which labels most protein classes, and wheat germ agglutinin (WGA) conjugated with Oregon Green (Invitrogen, Paisley, UK), which stains N-acetyl-D-glucosamine residues. The biofilm was stained with SYPRO and WGA dyes according to the manufacturer’s instructions, and fluorescence emission was measured using an SP8 CLSM (Leica, Wetzlar, Germany) and detected using the following laser excitation and emission wavelengths: 405 nm/655–755 nm for SYPRO and 459 nm/505–540 for WGA.

### 2.5. Visualization of Biofilms by Scanning Electron Microscopy (SEM)

SEM analysis was used initially to visualize the generated biofilms at different time points: 24 h, 48 h, and 72 h. The biofilms were grown on sterile glass coverslips with a diameter of 5 mm (Bar-Naor, Petah Tikva, Israel). The coverslips were removed, washed twice with sterile DDW, and fixed using 4% formaldehyde for 20 min. The samples were then washed again using DDW. The SEM analysis was performed using a Quanta 200 Environmental High-Resolution Scanning Electron Microscope (EHRSEM, FEI, Eindhoven, The Netherlands) [24].

### 2.6. Whole-Genome Sequencing

Whole-genome sequencing (WGS) of strain GBY4178 was performed by Illumina sequencing in a MiSeq (Illumina, San Diego, CA, USA) apparatus by outsourcing at the Weizmann Institute G-INCPM core facilities. DNA was extracted from fresh bacterial growth using Qiagen DNeasy Blood & Tissue Kit following the protocol for Gram-positive bacteria as per the manufacturer’s instructions (Qiagen, Hilden, Germany). Genomic libraries of 2 × 250 bp were prepared following mechanical shearing of DNA in a Covaris apparatus. Reads were quality-trimmed with BBDuk (sourceforge.net/projects/bbmap/, accessed 1 June 2018) and de-novo assembled with SPAdes v. 3.12.0 [25]. The genome was polished with Pilon [26] and annotated with Prokka [27]. The WGS of strain GBY4178 was performed as part of a larger study about coagulase-negative staphylococci causing mastitis in dairy animals in Israel. The first version of this genome is available at the NCBI. This Whole Genome Shotgun project has been deposited at DDBJ/ENA/GenBank under the accession JASFZT000000000. The version described in this paper is version JASFZT010000000.

### 2.7. RNA Extraction and Real-Time Reverse Transcriptase PCR (RT-qPCR)

The expression of genes involved in PIA production was quantified via RT-qPCR. Careful washing of biofilms generated at different time points was performed twice using PBS. The RNA extraction was performed as previously reported [28]. Briefly, 1 mL of RNA protect solution (Qiagen, Hilden, Germany) was added to each well, and the plate was incubated at room temperature for 5 min. The biofilms were dislodged by scraping and placed in 2 mL tubes, which were incubated at room temperature for 5 min. The samples were then centrifuged at 5000 rpm for 5 min. The RLT buffer (Qiagen) and 70 mg glass beads (Sigma Aldrich, Burlington, MA, USA) were added to biofilm pellets, which were subsequently disrupted with a FastPrep Cell Disrupter (MP Biomedicals, Santa Ana, CA, USA) in cycles of 45 s disruption and 5 min incubation on ice for three times. Isolation of the DNA-free total RNA was conducted using an RNeasy MINI kit (Qiagen) according to the manufacturer’s recommendations. The purity and concentration were determined using the Nanodrop-2000 Instrument (Thermo Fisher Scientific, Waltham, MA, USA).

Reverse transcription reaction was performed using 1 µg of total RNA as a template and the qScript cDNA Synthesis Kit (Quantabio, Beverly, MA, USA) according to the manufacturer protocol. The cDNA samples were stored at −20 °C until use. The *S. caprae* gene-specific primers were designed and manufactured by IDT using the Primer3 NCBI software (https://www.ncbi.nlm.nih.gov/tools/primer-blast/, accessed on 1 July 2018), with the genome of strain GBY4178 as a template, and aiming for PCR products up to 200 bp long (Table 1). For each primer set, a standard amplification curve was plotted (critical threshold cycle against the log of concentration), and only those with a slope ≈ −3.5 were considered reliable primers. The critical threshold cycle (Ct) was defined as the cycle in which fluorescence became detectable above the background fluorescence and was inversely proportional to the logarithm of the initial number of template molecules. The RT-qPCR reaction was performed as described previously [29]. Briefly, the RT-qPCR reaction mixture (15 μL) contained 7.5 μL PerfectCTa SYBER Green FastMix (Quantabio, Beverly, MA, USA), 1.75 μL of sterile distilled water, 5 μL of the cDNA sample, and 0.25 μM of the appropriate forward and reverse PCR primers. PCR conditions included an initial denaturation at 95 °C for 15 min, followed by a 40-cycle amplification that consisted of denaturation at 95 °C for 10 s, annealing at 58 °C for 20 s, and extension at 72 °C for 20 s. The RNA samples without reverse transcriptase were used as negative controls to determine the presence of residual genomic DNA in the RNA samples. The expression levels of the tested genes were normalized using the *gyrA* gene of *S. caprae* as an internal standard. The primers used for RT-qPCR are listed in Table 1.

### 2.8. Statistical Analysis

The statistical analysis consisted of *t*-test with a significance threshold set at *p* < 0.05. The results were obtained from three biological replicates, each performed in duplicate.

## 3. Results

### 3.1. Phenotypic Characterization of Biofilm Formation by S. caprae

**Figure 1:**
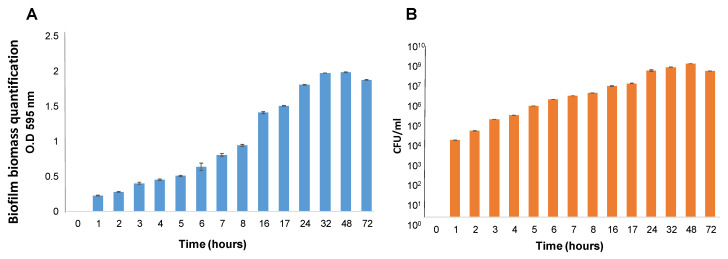
(**A**). Quantification using Crystal Violet staining. The biofilms were formed using TSB supplemented with 2.5% lactose and measured at different time points: 0 h–8 h, 16 h, 17 h, 24 h, 32 h, 48 h, and 72 h. Results show a readout of OD_595 nm_ following removing the medium and planktonic unattached cells, biofilm washing, and Crystal Violet staining. (**B**). Quantification using CFU assay. Biofilms were generated and washed as above. CFU counting was used to quantify viable bacteria at different time points: 0 h–8 h, 16 h, 17 h, 24 h, 32 h, 48 h, and 72 h.

The biofilm formation process by *S. caprae* was characterized stepwise to develop perceptions regarding the biofilm type and its morphology. The most crucial steps, bacterial adhesion and initial colonization onto surfaces, were investigated in a time-course developmental experiment. Results indicated that following a few hours of growth, the cells of *S. caprae* could adhere to polystyrene surfaces and start to accumulate, leading to the formation of substantial biofilm (Figure 1A,B). The progression of biofilm formation was tracked through bacterial biomass quantitation at various time points (Figure 1). Apparently, the peak in biofilm biomass accumulation was reached at 48 h, followed by a slight reduction in biomass after 72 h of growth.

**Figure 2:**
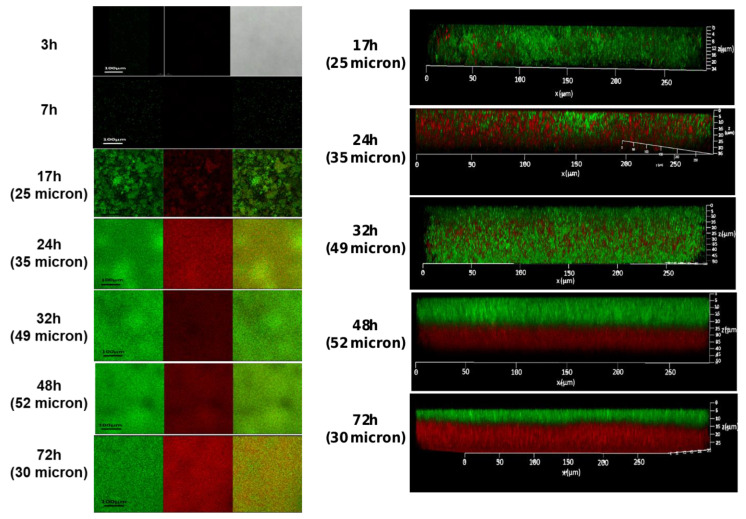
The CLSM images of *S. caprae* biofilms following live/dead staining. Biofilms were generated using TSB supplemented with 2.5% lactose and incubated at 37 °C for different times: 3 h, 7 h, 17 h, 24 h, 32 h, 48 h, and 72 h. Live bacteria were stained by SYTO 9 dye (green), and dead bacteria by PI dye (red) dye. Biofilm thickness as a measure of biomass is shown in brackets.

Confocal laser scanning microscopy (CLSM) analysis was used to visualize the robustness and vitality of the formed biofilms at different time points. Our observations indicated that bacterial cells start adhering to the polystyrene surface following 7 h of incubation. A notable increase in biofilm biomass (25 microns) was observed after 17 h of incubation, which was further increased at the following time points: 24 h (35 microns), 32 h (49 microns), and 48 h (52 microns) (Figure 2). Interestingly, following 72 h of growth, the biofilm biomass appeared to be less confluent, with some reduction in biofilm biomass depth (30 microns) and an increase in the presence of dead bacterial cells (Figure 2). In an attempt to improve biofilm cell viability through a possible decrease in the content of dead cells within the biofilm, we conducted an experimental setup by renewing the growth medium (that could increase the cell’s vitality during biofilm formation). Interestingly, such an experimental setup indeed increased bacterial viability. On the other hand, it significantly decreased biofilm biomass (to 10 microns), as observed using confocal microscopy following 72 h of biofilm growth. This result could indicate the developmental role of dead cells existing within the biofilm biomass during its maturation.

**Figure 3:**
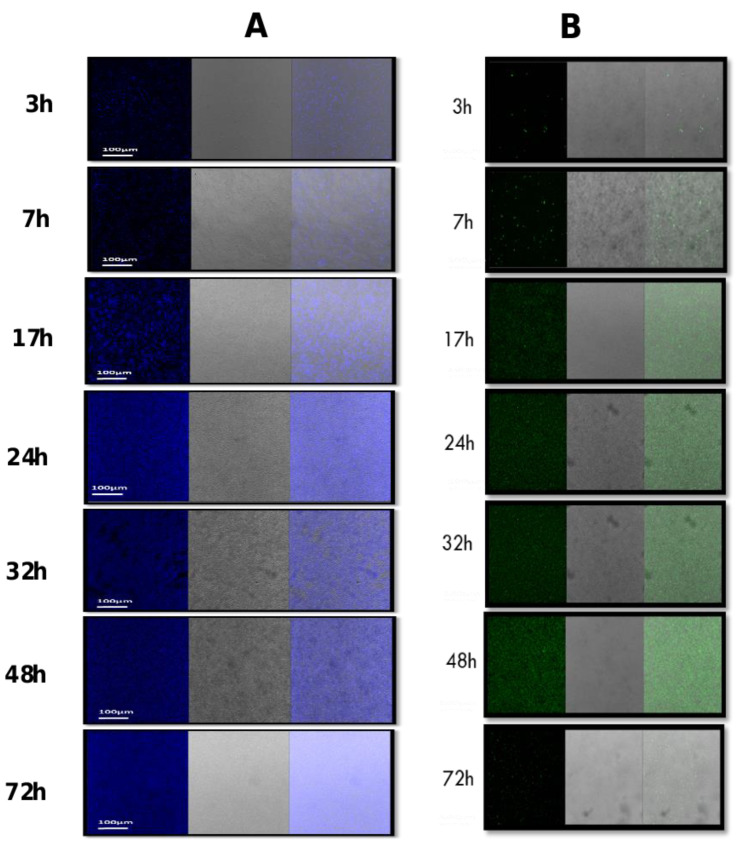
Composition of the biofilm matrix formed by *S. caprae*. The composition of biofilms at different time points was observed by CLSM after staining by two types of dyes: (**A**) FilmTracer SYPRO Ruby Biofilm Matrix stain, which labels most classes of proteins; (**B**) Wheat germ agglutinin (WGA) conjugated with Oregon Green, which stains N-acetyl-D-glucosamine residues.

The CLSM analysis was further used in conjunction with two different fluorescent dyes (WGA and SYPRO) to differentiate bacterial cells based on the possibility of producing polysaccharides or/and proteins within the biofilm matrix. Results showed that bacterial cells stained with SYPRO expressed matrix-associated proteins at all tested time points (Figure 3) culminated between 24 and 48 h of growth. Using WGA, the production of matrix-associated polysaccharides was observed at 3 h, 7 h, 24 h, 32 h, and 48 h. However, at 72 h, there was a relatively low amount of polysaccharides remaining in the biofilm matrix (Figure 3).

**Figure 4:**
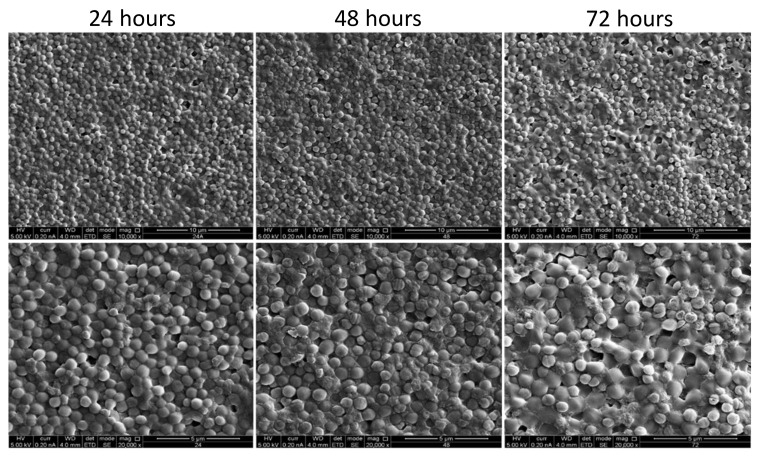
Scanning electron microscopy (SEM) images of the biofilm formed by *S. caprae*. SEM analysis of *S. caprae* was performed for biofilms developed on glass slides during incubation at 37 °C for different time points: 24 h, 48 h, and 72 h. The cells were observed and the images were taken at either ×10,000 (**upper panel**) or ×20,000 (**lower pannel**) magnifications.

The morphology of the developed biofilms was further characterized through scanning electron microscopy (SEM) observations of the biofilms generated at 24 h, 48 h, and 72 h. The SEM analysis confirmed the formation of the stable and confluent biofilm, with an accumulation of polysaccharides around the cocci cells, especially at 48 h (Figure 4).

### 3.2. Upr Egulation of the ica Operon during Biofilm Formation by S. caprae

**Figure 5:**
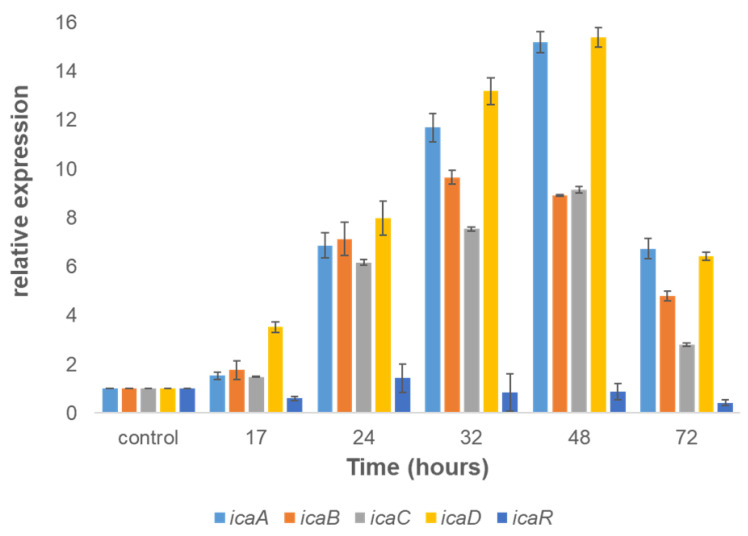
Dynamics of expression of the *ica* operon involved with biofilm formation by *S. caprae*. The relative expression of *icaA, icaB, icaC, and icaD* in biofilms was quantified by RT-qPCR at different times using the housekeeping gene *gyrA* for normalization and surface-unattached cells as a control.

The formation of biofilms depends on the synthesis of an extracellular matrix, which in *Staphylococcus* species is specified by the *ica* operon. Thus, it was hypothesized that the expression of the *ica* operon should be upregulated during biofilm formation, similarly to other staphylococci. Our results showed that the transition of *S. caprae* cells to a surface-associated state was accompanied by notable changes in the *icaABCD* operon regulation, especially during the maturation stages of biofilm development. The expression of these genes was significantly upregulated at the following time points: 24 h (7-fold), 32 h (12-fold), and 48 h (15-fold) compared to control (surface-unattached cells) samples (Figure 5).

## 4. Discussion

The biofilm-forming pathogenic bacteria that typically colonize the teat canal surface often facilitate the development of udder mastitis, which may subsequently affect the quality and safety of different dairy products [30]. Since biofilm formation represents a protective growth mode, it is regarded as an essential survival strategy for many pathogenic species in unfavorable environments [31]. Therefore, understanding the mechanism of biofilm formation by those pathogens can pave the way towards developing novel treatments for mitigating clinical and sub-clinical mastitis. Current thinking in the field suggests that biofilm formation is initiated in response to environmental signals and is dependent on the synthesis of an extracellular matrix that structures constituent cells together [32,33]. Many pathogenic species, including those responsible for acute mastitis, such as *S. aureus*, *E. coli*, and *S. uberis*, often form robust biofilms that could account for their persistence during milking or subsequent disinfection procedures in dairy parlors [34,35,36]. Thus, it is conceivable that the biofilm lifestyle could play a crucial role during bacterial infections, providing defense against the host immune system and resistance to antimicrobial treatments [37,38]. Consequently, one of the explanations regarding antibiotic resistance has been associated with biofilm-forming bacteria since antibiotic therapy often fails against biofilm-associated infections and requires high dosed antibiotic treatment for extended periods.

Coagulase-negative staphylococci (CoNS) are a group of bacterial species classified as either minor mastitis pathogens or commensal microbiota. Until recently, it was challenging to draw consistent conclusions about the significance of CoNS in bovine and small ruminants’ udder health. Some studies viewed CoNS as true mastitis pathogens, mainly retrieved from subclinical mastitis cases [39], while others considered CoNS to be commensal bacteria with limited or absent negative effects on SCC, milk quality, and milk production [40]. However, it has been lately acknowledged that CoNS may play a vital role in establishing the cow’s microbiome and have specific antibacterial activities during competition against pathogenic strains [41,42].

In the current study, we investigated the biofilm formation ability of one of the major CoNS—*S. caprae*, which may account for the prevalence of this bacterium in dairy goats. We examined the dynamics of biofilm formation at different time points using comprehensive phenotypic characterization. Our results confirmed the research assumption that the biofilm formation process of *S. caprae* is characterized by significant adherence of bacterial cells to the surface at the initial stages of biofilm development. This assumption was further reinforced using Cryo-SEM Microscopy, demonstrating the robustness of the encapsulated *S. caprae* cells within self-produced extracellular polymeric substances (Supplementary Figure S1). Following initial aggregation and a notable increase in biofilm biomass (within the first 24 h of growth), we observed biofilm maturation between 24–48 h of growth. At 72 h, we detected a decrease in the biofilm biomass, indicating the beginning of the biofilm dissociation process. In an attempt to improve biofilm biomass, we experimented with renewing the growth medium that could increase the biofilm cell’s vitality during biofilm formation. Remarkably, such an experimental setup indeed increased bacterial viability within the biofilm but, at the same time, significantly decreased biofilm biomass. We interpreted this result as a developmental role of the existence of dead cells within the mature biofilm.

Using WGA staining, combined with confocal microscopy analysis, we could detect PIA in the biofilm formed by *S. caprae*. PIA has been previously identified as the main exopolysaccharide component of the staphylococcal biofilm matrix [20]. The detection of PIA in *S. caprae* biofilms confirms its involvement in the biofilm formation process. Because PIA production is highly dependent on the presence and functionality of the *ica* operon in other *Staphylococcus* species, we hypothesized regarding its regulation during biofilm formation by *S. caprae*. Our gene expression analysis shows that the *icaADBC* operon indeed plays a role in biofilm formation by this bacterium. Thus, our results clearly demonstrate that all of the tested genes from this operon are notably upregulated and correlate to the dynamics of biofilm formation and maturation by *S. caprae*. It is conceivable, therefore, that the *ica* operon plays an essential role in regulating biofilm formation in *S. caprae*, similarly to other pathogenic *Staphylococcus* species.

Overall, the biofilm formation process characterized by significant upregulation in matrix-associated proteins and polysaccharides suggests the capacity of *S. caprae* for successful intramammary colonization, similar to other pathogenic and persistent *Staphylococcus* species.

## 5. Conclusions

This study aimed to characterize the process of biofilm formation by *S. caprae*, a pathogen associated with chronic mastitis in goats and sporadic infections in humans. The results showed that the dynamics of expression of the *ica* operon correlated to the biofilm formation process, suggesting that the mechanism of biofilm formation in this bacterium is overall similar to other *Staphylococcus* species. Moreover, expression dynamics of matrix-associated proteins and polysaccharides during biofilm maturation reinforces biofilm robustness and the capacity of *S. caprae* to colonize appropriate ecological niches.

## Figures and Tables

**Table 1:** List of primers designed for the RT-qPCR amplification.

Amplicon Size	Primer Sequence	Primer Designation	Gene
111	5′-TGCGAGCTTAATCGGATGTATTA-3′	icaA-F	*icaA*
	5′-TAGCCGACTTCTTTCAGTGC-3′	icaA-R	*icaA*
176	5′-TCGTACAGGCCTTTGGGATTT-3′	icaB-F	*icaB*
	5′-AAGGGTAAGCGATGGCATGT-3′	icaB-R	*icaB*
183	5′-TGCGCACACTTTAAAACCATGT-3′	icaC-F	*icaC*
	5′-ACCAATGCAGATGCCGAGAA-3′	icaC-R	*icaC*
126	5′-TCGCGATATCTTGCGCATTTT-3′	icaD-F	*icaD*
	5′-CTACGTTCTCCACATTGAGTGC-3′	icaD-R	*icaD*

## Data Availability

Data available upon request. The WGS of strain GBY4178 was performed as part of a larger study about coagu-lase-negative staphylococci causing mastitis in dairy animals in Israel. The first version of this genome is available at the NCBI. This Whole Genome Shotgun project has been deposited at DDBJ/ENA/GenBank under the accession JASFZT000000000. The version described in this paper is version JASFZT010000000.

## Supplementary Materials

The following supporting information can be downloaded at: https://www.mdpi.com/article/10.3390/microorganisms11061533/s1, Figure S1 (Cryo-SEM imaging of surfaced adhered biofilm formation by *S. caprae*).
