# Supplementary material for: Upregulation of ica Operon Governs Biofilm Formation by a Coagulase-Negative Staphylococcus caprae
Source: Microorganisms. 2023 Jun 9;11(6):1533. doi: 10.3390/microorganisms11061533 (PMC10305091; doi:10.3390/microorganisms11061533)

Supplementary Material for manuscript titled:

Upregulation of *ica* Operon Governs Biofilm Formation by a Coagulase-Negative *Staphylococcus caprae*

Hilla Oknin [1,2], Yulia Kroupitski [1], Moshe Shemesh [1,*] and Shlomo Blum [2,*]

[1] Institute for Postharvest Technology and Food Sciences, Department of Food Science, Agricultural Research Organization, Volcani Institute, Rishon LeZion 7505101, Israel

[2] Department of Bacteriology and Mycology, Kimron Veterinary Institute, Rishon LeZion 7534503, Israel

* Correspondence: moshesh@agri.gov.il (M.S.); shlomobl@moag.gov.il (S.B.)

Supplelentary Figure S1: Cryo-SEM imaging of surfaced adhered biofilm formation by S. caprae following 24h incubation.

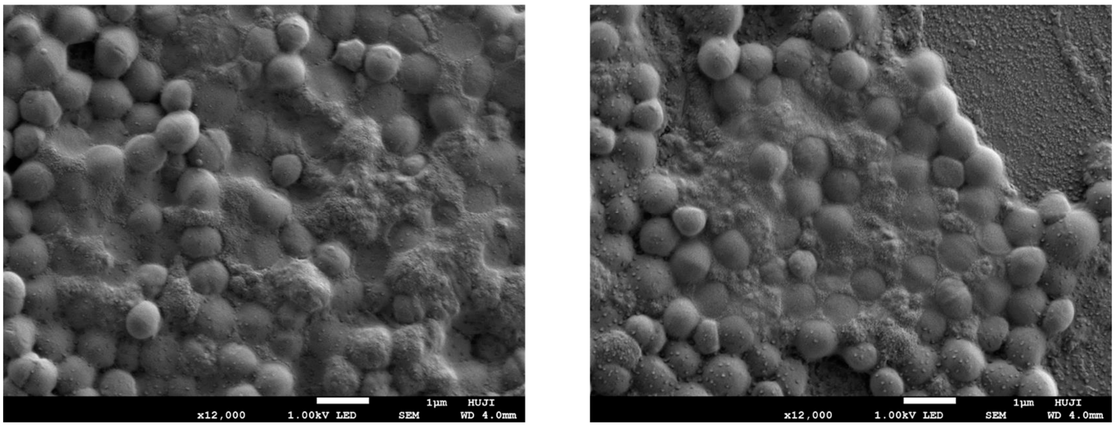

Supplement: Supplementary file 1 [file microorganisms-11-01533-s001.zip › microorganisms-2240124-supplementary.pdf]
